# SARS-CoV-2 antibody and neutralization dynamics among persons with natural- and vaccine-induced exposures

**DOI:** 10.1371/journal.pone.0331212

**Published:** 2025-09-03

**Authors:** Christopher S. Semancik, Romain Fantin, Julia Butt, Arturo Abdelnour, Viviana Loria, Carolina Porras, Amada Aparicio, Sarah S. Jackson, Roy Wong-McClure, Rebeca Ocampo, Melvin Morera, Michael Zúñiga, Alejandro Calderón, Bernal Cortés, Roberto Castro, Marco Binder, Tim Waterboer, D. Rebecca Prevots, Rolando Herrero, Allan Hildesheim, for the RESPIRA Study Group

**Affiliations:** 1 Epidemiology and Population Studies Section, National Institute of Allergy and Infectious Diseases, National Institutes of Health, Rockville, Maryland, United States of America; 2 Agencia Costarricense de Investigaciones Biomédicas-Fundación INCIENSA (ACIB-FUNIN), San José, Costa Rica; 3 Infections and Cancer Epidemiology, German Cancer Research Center, Heidelberg, Germany; 4 Caja Costarricense del Seguro Social, San José, Costa Rica; 5 Infections and Immunoepidemiology Branch, Division of Cancer Epidemiology and Genetics, National Cancer Institute, Bethesda, Maryland, United States of America; 6 Ministerio de Salud, San José, Costa Rica; 7 Research Group, “Dynamics of Early Viral Infection and the Innate Antiviral Response”, Division of Virus-Associated Carcinogenesis (D430), German Cancer Research Center, Heidelberg, Germany; Janssen Vaccines & Prevention B.V., NETHERLANDS, KINGDOM OF THE

## Abstract

Previous SARS-CoV-2 research indicates that antibody levels and corresponding neutralization potential increase with additional exposures (comprising vaccination or infection), and that hybrid immunity resulting from combined vaccination and natural infection is more robust than either alone. However, it is unclear whether or how antibody levels increase or eventually plateau with repeated exposures and how SARS-CoV-2 exposure differs by sex or other demographic factors. Research regarding the association of antibody production with neutralization potential is also limited. We conducted this analysis within the RESPIRA population-based cohort in Costa Rica to investigate relationships between antibody levels and neutralization potential at increasing exposure levels. We examined immunological profiles from systematically defined single-exposure groups (one vaccine dose or one natural infection), double-exposure groups (two vaccine doses or one vaccine dose following a natural infection), and a triple-exposure group (two vaccine doses following a natural infection). We used a S1-RBD-based serological assay for antibody level detection and a pseudovirion assay for neutralization potential quantification. Using linear regression, we compared antibody levels and pseudoneutralization geometric mean titers between exposure groups. For single exposure groups, one vaccine dose was inferior to natural infection, but a second vaccine dose was superior to natural infection. For double exposure groups, those who were vaccinated once after infection developed higher levels of antibodies and higher neutralization potential compared with those who had only two vaccine doses. We note that peak antibody levels following an exposure may plateau after two exposures while neutralization potential continues to increase with a third exposure dose. Response patterns were comparable in males and females and in sensitivity analyses stratified by age, vaccine type, and pandemic wave. These results provide evidence that SARS-CoV-2 vaccination after COVID infection provides immunological benefit and suggest neutralization potential continues to increase after a second vaccine dose despite plateauing of antibody levels.

## Introduction

Severe acute respiratory syndrome coronavirus 2 (SARS-CoV-2) infection has caused substantial morbidity and mortality worldwide, and recent estimates suggest that the death toll associated with this virus may have been as high as 18 million in 2020–2021 [1]. Since the pandemic began, the SARS-CoV-2-related immune response in humans has been elucidated [2,3], and several effective coronavirus disease (COVID-19) vaccines were developed, including a widely used vaccine platform based on the receptor-binding domain (RBD) of the S1 subunit of SARS-CoV-2 [4–6].

Published work indicates that 1) antibody levels and accompanying neutralization potential increase with increasing number of vaccination doses in the initial vaccination series [7–11]; 2) humoral immunological responses induced through a two-dose vaccine series are stronger than those observed following a natural infection [12–14]; and 3) antibody levels and immunological responses induced through hybrid immunity (infection plus vaccination) are more robust than those observed with either vaccination or natural infection alone [10,11,15–24].

While the patterns summarized above are well established, the effect of continued boosting on peak responses remains unclear [25,26], specifically whether patterns observed in antibody levels and the more functionally relevant neutralization potential parallel each other with increasing number and types of exposure (i.e., vaccination vs. infection) [27,28].

Data are also lacking on whether immune responses to SARS-CoV-2 infection or vaccination differ in males and females. More generalized immunological work (not specific to COVID) has suggested that sex plays a role in the immune response to viral exposure, with females having been shown to develop higher levels of pathogen-specific antibodies than males following infection [29]. Whether this phenomenon is observed following SARS-CoV-2 infection or vaccination is not well understood, as a recent publication shows that males have an initially higher antibody response while responses in females were more tightly linked to neutralization potential [30].

Considering these observations, the present study had three central objectives. First, we aimed to confirm that immune response increases with increasing exposure and that a combination of infection and vaccination leads to more robust responses, given a comparable number of exposures. Second, we aimed to better understand the relationship between antibody levels (a measure of antibody quantity) and neutralization potential (a more direct measure of antibody function achieved with affinity maturation), given different types and numbers of exposures. Third, we aimed to examine whether observed responses differ between males and females. To achieve these objectives, we evaluated serum antibody responses and neutralization potential among participants in a population-based cohort study of SARS-CoV-2 in Costa Rica named the RESPIRA cohort [31].

## Materials and methods

### Study population

The parent cohort from which samples for the present study were selected, the RESPIRA cohort, has been previously described [31]. In brief, RESPIRA recruited and followed for two years 999 confirmed COVID-19 cases identified through the Costa Rica national COVID surveillance registry and 1,999 community controls matched to cases on age, sex, and geography. Participants were recruited into the cohort between November 2, 2020 and September 4, 2021. Cases had been diagnosed from March 2020 through July 2021 during the first (ancestral strain) and second (alpha and gamma variants) waves of the pandemic in Costa Rica. In Costa Rica, vaccination against COVID-19 started in December 2020 beginning with those over 58 years old, health personnel, and high-risk groups, and then extended to other ages and segments of the population over time [32]. As part of the main cohort, all enrollment specimens and a subset of follow-up specimens from cases and controls were tested for SARS-CoV-2 antibodies, as described below.

Within the cohort defined above, for the present analysis, we identified 684 individuals with serum samples collected from 30–89 days after SARS-CoV-2 infection or a SARS-CoV-2 vaccine (i.e., during peak immune response period following an exposure) [30,33], as follows (Table 1):

**Table 1:** Study Exposure Group Classification.

Study Group	Description	N (Individuals with Luminex 1:1000 S1-RBD Result)	N (Individuals with Pseudovirus Neutralization Result)
Natural Infection Only (NI)	Individuals who were infected before any vaccine doses with sample collected 30–89 days post-natural infection	109	48
Vaccination Only Post Dose 1 (VAX-1)	Individuals vaccinated before infection with sample collected 30−89 days post-1^st^ dose (and before 2^nd^ dose)	128	38
Vaccination Only Post Dose 2 (VAX-2)	Individuals vaccinated before infection with sample collected 30−89 days post-2^nd^ dose (and before 3^rd^ dose)	83	17
Hybrid Immunity Post Dose 1 (HI-1)	Individuals vaccinated post-infection with sample collected 30−89 days post-1^st^ dose (and before 2^nd^ dose)	202	57
Hybrid Immunity Post Dose 2 (HI-2)	Individuals vaccinated post-infection with sample collected 30−89 days post-2^nd^ dose (and before 3^rd^ dose)	162	18
TOTAL		684	178

1) 109 individuals with one natural infection (NI; single exposure); 48 of these participants were selected for neutralization testing;
2) 128 individuals with one dose of vaccine (VAX-1; single exposure); 38 of these participants were selected for neutralization testing;
3) 83 individuals with two doses of vaccine (VAX-2; double exposure); 17 of these participants were selected for neutralization testing;
4) 202 individuals infected with SARS-CoV-2 once who subsequently received one dose of vaccine (HI-1; hybrid immunity double exposure); 57 of these participants were selected for neutralization testing; and
5) 162 individuals infected with SARS-CoV-2 once who subsequently received two doses of vaccine (HI-2; hybrid immunity triple exposure); 18 of these participants were selected for neutralization testing.

### Definitions of previous exposure

We considered a participant to have had a previous infection if, prior to their first dose of vaccine (if they received a vaccine), a participant met one of the following conditions:

1) Received a positive COVID diagnosis through a PCR-test (n = 447; 94.5%), epidemiological link (n = 7; 1.5%), or an official diagnosis without specification (n = 7; 1.5%); or
2) Was seropositive to SARS-CoV-2 antibodies at baseline without an official diagnosis (n = 12; 2.5%).

Vaccination status (including dates and number of doses) for participants was ascertained by an individual questionnaire at baseline, and by searching in their individual digital health record [31,34].

Study participants were classified into each exposure group using the case definitions and conditions outlined in Table 1.

### Sample collection and testing

Blood was collected as previously described [31]. Briefly, blood samples were collected by venipuncture into a red-top tube without preservative and stored in cold boxes between 2–10°C for up to 12 hours prior to centrifugation, serum aliquoting, and transfer to a −80°C freezer. Serum specimens remained frozen until testing was performed.

Testing for antibodies to SARS-CoV-2 was performed at the German Cancer Research Center (DKFZ Heidelberg) as described elsewhere [35]. To quantify antibody levels, recombinantly expressed S1-RBD protein of the SARS-CoV-2 proteome was linked to the bead surface of fluorescently labelled polystyrene beads (Luminex, Austin, Texas, USA). Antigen-loaded beads were incubated with serum sample (1:1000 serum dilutions). A Luminex 200 Analyzer (LuminexA) was used to identify the bead set and its respective antigen, and to quantify the amount of serum antibody bound to the antigen by reporter fluorescence as median fluorescence intensity (MFI) of at least 100 beads per antigen and sample measured.

A subset of 178 sera was selected based on age and case-control status to ensure even distribution across these groups (Table 1). This subset was tested for functional virus neutralization capacity. For this purpose, a lentivirus-based pseudovirus neutralization assay was employed using a codon-optimized SARS-CoV-2 (Wuhan Hu-1) spike protein for pseudotyping [36]. The pseudovirus encoded the firefly luciferase gene used for quantitative measurement of virus entry into ACE2-transduced A549 lung epithelial cells (ACE2). Briefly, pseudoparticles were incubated with a 10-step 2-fold dilution series of test sera for 1 hour at 37°C before being added to ACE2-A549 cells. Twenty-four hours later, cells were lysed and luciferase activity was measured. Luciferase signals were standardized to 0% (uninfected cells)–100% (cells infected with pseudovirus without serum) and plotted over serum dilution. The 50% inhibitory titer was determined by logistic regression. This assay has been validated previously by comparing defined sera against an independent authentic virus (SARS-CoV-2) [30,31].

### Statistical analysis

To analyze antibody levels by exposure group, the geometric mean levels from the Luminex assay (measured in MFI) and the geometric mean titers from the pseudovirus neutralization assay were calculated. Geometric means and accompanying 95% confidence intervals (CIs) were calculated using the *Gmean* function from the *DescTools* package in RStudio, Version 4.3.0. Then, to compare differences in geometric mean titers among groups, linear regression models were run on the logarithms to base 10 of the antibody levels and the neutralization titers. When comparing between study groups, we utilized regression p-values, as well as pairwise comparisons, to identify heterogeneity between groups. To ensure that our findings were not explained by differences between study groups with respect to age, vaccine type, and pandemic wave, analyses stratified by these factors were performed and reported (sensitivity analyses).

The analyses described above were also stratified by sex. We used interaction p-values from regression models including sex, study group, and an interaction term between sex and study group, to derive p-values that compared geometric mean level or titer ratios at each exposure level between males and females.

### Ethics statement

This study involves human participants and was approved by Comité Ético Científico Central (CEC-CENTRAL-CCSS): protocol # R020-SABI-00261. Informed, signed consent was obtained from all study participants or their parents (for participants younger than 18) to participate in the study before taking part, in the presence of a witness, as mandated by Costa Rican law.

## Results

### Demographic and clinical information by exposure group

This analysis was performed on a total of 684 participants tested for SARS-CoV-2 S1-RBD antibodies and a subset of 178 participants tested for SARS-CoV-2 neutralization potential. Demographic and clinical information for the 684 individuals included in our analyses is shown in Table 2, overall and by study group.

**Table 2:** Demographic and Other Characteristics of Study Population Overall and by Study Group.

Characteristic	Overall (n = 684)		HI-1 (n = 202)		HI-2 (n = 162)		VAX-1 (n = 128)		VAX-2 (n = 83)		NI (n = 109)	
	#	%	#	%	#	%	#	%	#	%	#	%
Sex												
Female	382	55.8	124	61.4	81	50.0	76	59.4	46	55.4	55	50.5
Male	302	44.2	78	38.6	81	50.0	52	40.6	37	44.6	54	49.5
Age												
<20	106	15.5	52	25.7	3	1.9	26	20.3	0	0.0	25	22.9
20-59	415	60.7	149	73.8	75	46.3	102	79.7	26	31.3	63	57.8
60+	163	23.8	1	0.5	84	51.9	0	0.0	57	68.7	21	19.3
Median [IQR]	43 [31]		32 [26]		60 [18]		31.5 [20.5]		62 [9.5]		36 [35]	
Disease severity*												
Mild	262	63.0	96	57.5	88	62.8	N/A	N/A	N/A	N/A	78	71.6
Moderate	136	32.7	66	39.5	46	32.9	N/A	N/A	N/A	N/A	24	22.0
Severe (hospitalization)	18	4.3	5	3.0	6	4.3	N/A	N/A	N/A	N/A	7	6.4
Vaccine Descriptor**												
mRNA Only	467	79.1	156	77.2	128	79.0	100	78.1	70	84.3	N/A	N/A
Non-mRNA Only	103	17.5	45	22.3	22	13.6	28	21.9	6	7.2	N/A	N/A
Mixed	20	3.4	1	0.5	12	7.4	0	0.0	7	8.4	N/A	N/A
# Days Between Most Recent Exposure and Sample Collection												
Median [IQR]	54 [26]		55 [24]		53 [24.8]		53.5 [27.3]		62 [25.5]		54 [22]	

* A total of 57 disease severity measurements that are missing. Of these 57, 35 are from the HI-1 group and 22 are from the HI-2 group. ** Of the participants that received a first vaccine dose, 453 (78.8%) received Pfizer, 116 (20.2%) received AstraZeneca/Oxford, 3 (0.5%) received Janssen, and 3 (0.5%) received Moderna. Of participants that received a second vaccine dose, 213 (86.9%) received Pfizer/BioNTech, 29 (11.8%) received AstraZeneca/Oxford, 2 (0.8%) received Moderna, and 1 (0.4%) received Sinopharm.

Participants were majority female (55.8%), with the proportion of female participants ranging from 50.0% (HI-2 group) to 61.4% (HI-1 group). The median age of participants was 43.0 years and ranged from 31.5 years (VAX-1 group) to 62.0 years (VAX-2 group). Overall, 84.1% of participants had at least one dose of vaccine. The younger average age of individuals in the VAX-1, HI-1, and NI groups are due to vaccination policies in Costa Rica that emphasized a three-week interval between doses in the early months after vaccine introduction, in the period during which older individuals were prioritized. As a result, individuals who were unvaccinated and/or who were sampled during the peak immune response period following initial vaccination (defined in this study as 30−89 days) tended to be younger than vaccinated individuals who were sampled during the peak immune response period following the second vaccination. The majority (79.1%) of vaccinated participants received messenger ribonucleic acid (mRNA) vaccines only and the majority (96.6%) of SARS-CoV-2 infected participants had mild (63.0%) or moderate (32.7%) disease severity that did not require hospitalization. Per design, the median number of days between most recent exposure and sample collection was 54.0 and was similar across study groups.

### Single exposure: Immunogenicity of natural infection compared to single vaccination

The lowest levels of COVID exposures in our cohort — single exposures — consist of a single natural infection with no history of vaccination (NI) and one dose of vaccine without any evidence of infection (VAX-1). We compared immune response at these varying single exposures to better understand how antibody production and neutralization potential differ between a natural infection and a single dose of vaccine.

**Fig 1:**
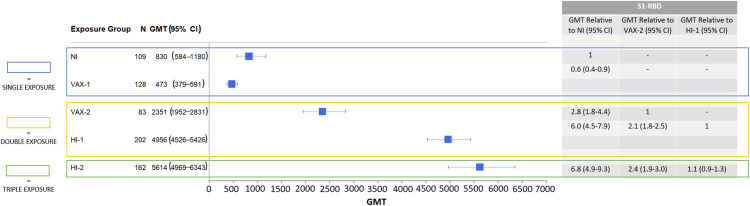
Forest Plot and Summary Table Comparing Overall Antibody Levels (S1-RBD) by Study Group.

Levels of S1-RBD antibodies after a single vaccination dose were associated with lower antibody levels compared to NI (geometric mean [GMT] ratio, 0.6, 95% CI = 0.4–0.9) (Fig 1). The GMT level of S1-RBD antibodies was 830 MFI (95% CI = 584−1180) for the NI group and 473 MFI (95% CI = 379−591) for the VAX-1 group (Fig 1).

**Fig 2:**
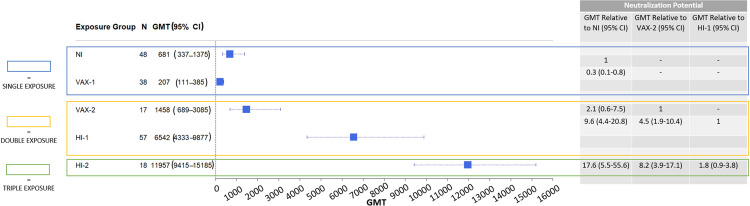
Forest Plot and Summary Table Comparing Overall Neutralization Potential by Study Group.

Pseudoneutralization titers among those in the VAX-1 group were also inferior to those observed following NI (GMT ratio = 0.3, 95% CI = 0.1–0.8) (Fig 2). The pseudoneutralization GMT titer was 681 (95% CI = 337−1375) for the NI group compared to 207 (95% CI = 111−385) for the VAX-1 group (Fig 2). These neutralization potential results are consistent with the modest levels of S1-RBD antibody production observed among individuals with a single exposure (natural infection or vaccination).

### Double exposure: Immunogenicity of natural infection plus vaccination and double vaccination compared to natural infection

We next examined individuals with two SARS-CoV-2 exposures. Specifically, this group comprised either two doses of vaccine (VAX-2) or one dose of vaccine and a prior natural infection (HI-1). When comparing these two groups to natural infection (shown to be the more immunologically robust of the single exposures), we aimed to understand the extent to which a second SARS-CoV-2 exposure affected immunological responses.

S1-RBD antibody levels were 2.8 times higher (95% CI = 1.8–4.4) in the VAX-2 group compared to the NI group (Fig 1). The GMT level of S1-RBD antibodies was 2351 MFI (95% CI = 1952–2831) for the VAX-2 group compared to 830 MFI in the NI group (95% CI = 584–1180) (Fig 1). S1-RBD antibody levels were also higher compared to the NI group (GMT ratio = 6.0, 95% CI = 4.5–7.9) in participants from the HI-1 group (Fig 1). The GMT level of S1-RBD antibodies was 4956 MFI (95% CI = 4526–5426) for the HI-1 group compared to 830 MFI in the NI group (95% CI = 584–1180) (Fig 1).

Pseudoneutralization titers were similarly high in both double exposure groups (VAX-2 and HI-1) as observed for S1-RBD antibody levels. Pseudoneutralization GMTs among those in the VAX-2 group were 2.1-fold higher compared to NI (95% CI = 0.6–7.5), although this relationship was not statistically significant (Fig 2). The pseudoneutralization GMT titer was 1458 (95% CI = 689–3085) for the VAX-2 group compared to 681 (95% CI = 337–1375) for the NI group (Fig 2). Similar to the increase in antibody levels, pseudoneutralization was also stronger in participants from the HI-1 group compared to the NI group (GMT ratio = 9.6, 95% CI = 4.4–20.8) than for when comparing the HI-1 group to the VAX-2 group (Fig 2). The pseudoneutralization GMT was 6542 (95% CI = 4333–9877) for the HI-1 group compared to 681 (95% CI = 337–1375) for the NI group (Fig 2).

### Double exposure: Hybrid immunity (natural infection plus single vaccination) compared to double vaccination

We also compared the two double exposure groups to one another directly to understand whether a combined exposure to SARS-CoV-2 (one infection and one vaccination) was more immunologically robust than double exposure of just one type (two vaccine doses). S1-RBD antibody levels were 2.1 times higher in the HI-1 group compared to the VAX-2 group (95% CI = 1.8–2.5) (Fig 1). The GMT level of antibodies was 4956 MFI (95% CI = 4526−5426) for the HI-1 group compared to 2351 MFI (95% CI = 1952−2831) for the VAX-2 group (Fig 1).

Like for S1-RBD antibody levels, neutralization potential showed a significant increase when comparing double exposures (VAX-2 and HI-1) to each other. Specifically, pseudoneutralization titers were 4.5 times higher among those in the HI-1 group compared to those in the VAX-2 group (95% CI = 1.9–10.4) (Fig 2). The pseudoneutralization GMT was 6542 (95% CI = 4333−9877) for the HI-1 group compared to 1458 (95% CI = 689−3085) for the VAX-2 group (Fig 2). This indicates that at double exposure, hybrid immunity with one vaccine dose leads to higher antibody levels and pseudoneutralization titers than those observed with two vaccine doses.

### Triple exposure: Impact of boosting hybrid immunity with a second vaccine dose

Next, we studied participants with a history of previous SARS-CoV-2 infection and two vaccine doses (HI-2 group) and compared them to individuals with a history of infection plus a single vaccination dose (HI-1 group) to determine the impact of boosting hybrid immunity.

S1-RBD antibody levels were comparable in the HI-2 and HI-1 groups (GMT ratio = 1.1, 95% CI = 0.9–1.3) (Fig 1). The GMT level of S1-RBD antibodies was 5614 MFI (95% CI = 4969−6343) for the HI-2 group compared to 4956 MFI (95% CI = 4526−5426) for the HI-1 group (Fig 1).

In contrast to what we observed for S1-RBD antibody levels, pseudoneutralization titers did not seem to plateau after two exposures in the hybrid immunity group. Pseudoneutralization GMTs were 1.8 times higher in individuals from the HI-2 group, compared to those from the HI-1 group (GMT ratio = 1.8), although the observed increase was not statistically significant (95% CI = 0.9–3.8) (Fig 2). The pseudoneutralization GMT was 11957 (95% CI = 9415−15185) for those in the HI-2 group compared to 6542 (95% CI = 4333−9877) for the HI-1 group (Fig 2). Taken together, this suggests that there may be a plateauing in peak antibody levels but suggests a continued increase in neutralization potential when boosting hybrid immunity.

### Exposure and response differences by sex

**Fig 3:**
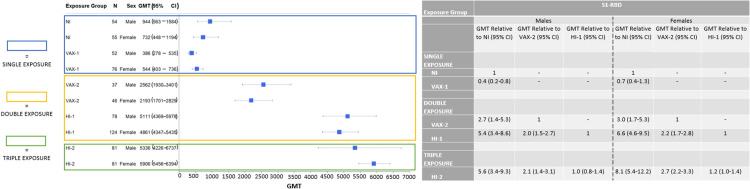
Forest Plot and Summary Table Comparing Sex-Stratified Antibody Levels (S1-RBD) by Study Group. None of the p-values comparing S1-RBD antibody GMT ratios between males and females were statistically significant. Interaction p-values comparing GMT ratios between groups are as follows: VAX-1:NI: 0.15; VAX-2:NI: 0.82; HI-1:NI: 0.48; HI-2:NI: 0.28; HI-1:VAX-2: 0.58; HI-2:VAX-2: 0.24; HI-2:HI-1: 0.32.

**Fig 4:**
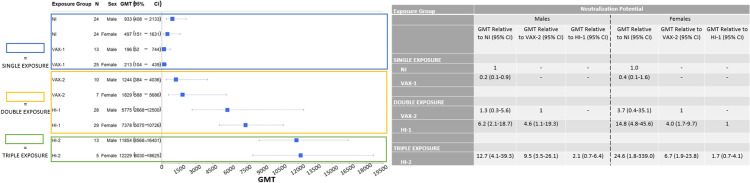
Forest Plot and Summary Table Comparing Sex-Stratified Neutralization Potential by Study Group. None of the p-values comparing neutralization potential GMT ratios between males and females were statistically significant. Interaction p-values comparing GMT ratios between groups are as follows: VAX-1:NI: 0.47; VAX-2:NI: 0.43; HI-1:NI: 0.27; HI-2:NI: 0.60; HI-1:VAX-2: 0.87; HI-2:VAX-2: 0.66; HI-2:HI-1: 0.80.

Next, we performed analyses stratified by sex, to determine whether the patterns reported above differed between males and females. Patterns observed in males and females were generally comparable and similar to those reported overall (Figs 3 and 4). No statistically significant differences were noted between S1-RBD levels and neutralization responses in males versus females. However, two suggestive differences were noted. The first was that the inferior overall neutralization potential observed after one vaccine dose compared to natural infection seemed to be influenced more strongly by the effect in males (GMT ratio among males = 0.2, 95% CI = 0.1–0.9; GMT ratio among females = 0.4, 95% CI = 0.1–1.6; p-heterogeneity = 0.47). The second was that the increase in overall neutralization potential observed after two vaccine doses compared to natural infection seemed to be influenced more strongly by the effect in females (GMT ratio among females = 3.7, 95% CI = 0.4–35.1; GMT among males = 1.3, 95% CI = 0.3–5.6; p-heterogeneity = 0.43).

### Sensitivity analyses

We performed sensitivity analyses restricted to ages 20–59 years, mRNA vaccine type, and stratified by pandemic wave (ancestral vs. alpha/gamma) to ensure that our overall findings were not explained by any of these factors. The patterns of S1-RBD levels and neutralization potential observed in these restricted analyses were comparable to those reported overall (S1 Tables in S7-S14).

## Discussion

In this report, we evaluated patterns of immune response to SARS-CoV-2 by number and type of exposure (natural infection and vaccination). We evaluated both antibody levels and the more functionally relevant neutralization potential, and compared patterns. This was done in an effort to understand the extent to which increasing exposures to SARS-CoV-2 affect immunological responses, particularly when combining vaccination and infection. We also examined patterns separately by sex, given suggestions that immune responses to viral infections and vaccination differ by sex [29]. Our main findings were that 1) a single vaccine dose is inferior to natural infection in terms of antibody production and pseudoneutralization titers, although two vaccine doses without infection (VAX-2) is superior to natural infection alone; 2) among individuals with two exposures, those who were infected followed by one vaccine dose (hybrid immunity) developed higher levels of antibodies, and higher neutralization potential than uninfected individuals who received two vaccine doses; and 3) among those infected and vaccinated, peak antibody levels following an exposure may plateau after two exposures while neutralization potential seems to continue to increase with a third exposure dose. We also observed largely comparable response patterns among males and females, although a few suggestive differences were noted, as discussed further below.

Our observation that a single vaccination dose leads to lower antibody production and neutralization potential than natural infection, and that a second vaccine dose given to a non-infected individual reverses this pattern is in line with previous reports [25,37] and consistent with the expectation that exposure to a single antigenic stimulus (vaccination) would lead to lower antibody production than the more prolonged (days to weeks) exposure to virus particles that occur with a natural infection. A 2022 study by Naranbhai and colleagues reported that previously uninfected individuals who received a single vaccine dose exhibited significantly lower antibody concentrations than those who were previously infected and had recovered [37]. Furthermore, a study by Srivastava and colleagues showed that individuals who had been previously infected reached a pre-specified antibody level cutoff point before those who had only had one vaccine dose [25].

Our finding that infection plus one vaccine dose (hybrid immunity) leads to a more robust immune response than two vaccine doses is also supported by previous studies [38–40]. Other studies have also shown higher levels of neutralizing antibodies in those vaccinated individuals that have already been infected with SARS-CoV-2, compared to vaccinated individuals that have not been infected. However, our study is unique in that it compares neutralizing antibody levels between naturally infected, single-vaccinated (not yet fully vaccinated) individuals to double-vaccinated, non-infected individuals, and in that it compares functional neutralization potential between such groups.

Another notable finding from our study is that triple exposure (infection followed by two vaccine doses) led to suggestively stronger and more robust neutralization potential than double exposure (infection followed by one vaccine dose), despite the suggestion that peak antibody levels may plateau at these higher exposure levels. While it is possible that this observation is explained by a saturation effect of the S1-RBD binding assay at high antibody levels, the finding is consistent with continued affinity maturation with an increasing number of exposures in the absence of continued increases in antibody production. Specifically, this disproportionate increase in neutralization potential associated with hybrid immunity suggests that B cell affinity maturation leads to higher viral neutralization potential which is more robust in the context of natural infection than in vaccination alone [41]. Such affinity maturation processes and effects can often be observed when somatic hypermutations occur in immunoglobulin genes in B cells, along with simultaneous selection for antigen binding [42,43]. Similarly, there may exist an immunological exhaustion process by which the production of antibodies slows to a plateau, but the response of existing antibodies improves through affinity maturation [44].

Due to data on other pathogens suggesting differences in immune response by sex [29], we examined immune response to SARS-CoV-2 separately in males and females. Here, we observed similar patterns for males and females, with no significant differences observed. However, a few suggestive differences were seen that might merit further attention. The first suggestive difference of note is that males generally had inferior antibody and neutralization levels following a single vaccine dose compared to natural infection, which contributed to this overall observed effect in the entire population. Another important suggestive difference is that neutralization potential achieved after two vaccine doses in males was comparable to that noted following natural infection, while females who received two vaccine doses had 3.5-fold increased levels of neutralization potential compared to those who were naturally infected. This observation suggests that females have more efficient immune responses with increasing SARS-CoV-2 exposures than males, which is consistent with SARS-CoV-2 immune response differences by sex in a mouse model [45]. These observed sex differences merit further examination in larger human population samples.

Our study has multiple strengths. By using a serological assay and a pseudovirion neutralization assay, we could evaluate patterns of S1-RBD antibody production and neutralization potential in parallel. In addition, our study was embedded in a large, population-based cohort in which exposure groups were systematically defined and where timing between exposure and blood collection was carefully timed to correspond to the window of time when peak responses are expected. Furthermore, we were able to limit to the period of 30–89 days since exposure, which has previously exhibited the strongest antibody responses with no significant differences in responses over this window [30,33]. This allows more consistent, less varied interpretations of antibody response with respect to time-since-exposure. Our study also has a few limitations. The number of participants included in some of our analyses was modest, meaning that some differences, particularly those stratified by sex, might have been missed or understated. Given the complexity and cost of performing the functional neutralization potential assay, we were able to perform this assay on only a subset of participants. In addition, the early prioritization in Costa Rica of vaccination among the elderly during a time when interval between doses was short led to imbalances across our study groups with respect to age. While these imbalances could have introduced confounding, our sensitivity analyses suggest that they did not. Finally, because cases were diagnosed from March 2020 through July 2021 (during ancestral, alpha, and gamma variants), we do not have data on later variants, such as delta and omicron.

In summary, we have confirmed that increasing exposures increase the robustness of corresponding immune response, that hybrid immunity results in a more robust immune response than vaccination alone, and that antibody levels might plateau at high exposure levels accompanied by continued increases in neutralization potential.

## Supporting information

Table S1 Semancik et al Supplementary Materials Revised 7152025. (DOCX)
