# Supplementary material for: SARS-CoV-2 antibody and neutralization dynamics among persons with natural- and vaccine-induced exposures
Source: PLoS One. 2025 Sep 3;20(9):e0331212. doi: 10.1371/journal.pone.0331212 (PMC12407447; doi:10.1371/journal.pone.0331212)
Supplement: Table S1 — (DOCX) [file pone.0331212.s001.docx]

Supplementary Materials:

Table S1: Overall Antibody Levels (S1-RBD) by Study Group (for Those with 1:1000 Dilutions):

| Study Group | S1-RBD | | | | | | | |
| --- | --- | --- | --- | --- | --- | --- | --- | --- |
|  | N | GMT | GMT Relative to NI | 95% CI | GMT Relative to VAX-2 | 95% CI | GMT Relative to HI-1 | 95% CI |
| SINGLE EXPOSURE |  |  |  |  |  |  |  |  |
| Infection | 109 | 830 | 1.0 | - | - | - | - | - |
| Vaccination - 1 dose | 128 | 473 | 0.6 | 0.4-0.9 | - | - | - | - |
|  |  |  |  |  |  |  |  |  |
| DOUBLE EXPOSURE |  |  |  |  |  |  |  |  |
| Vaccination - 2 doses | 83 | 2351 | 2.8 | 1.8-4.4 | 1.0 | N/A | - | - |
| Infection + Vaccination - 1 dose | 202 | 4956 | 6.0 | 4.5-7.9 | 2.1 | 1.8-2.5 | 1.0 | N/A |
|  |  |  |  |  |  |  |  |  |
| TRIPLE EXPOSURE |  |  |  |  |  |  |  |  |
| Infection + Vaccination - 2 doses | 162 | 5614 | 6.8 | 4.9-9.3 | 2.4 | 1.9-3.0 | 1.1 | 0.9-1.3 |

Table S2: Overall Neutralization Potential by Study Group:

| Study Group | Pseudoneutralization | | | | | | | |
| --- | --- | --- | --- | --- | --- | --- | --- | --- |
|  | N | GMT | GMT Relative to NI | 95% CI | GMT Relative to VAX-2 | 95% CI | GMT Relative to HI-1 | 95% CI |
| SINGLE EXPOSURE |  |  |  |  |  |  |  |  |
| Infection | 48 | 681 | 1.0 | N/A | - | - | - | - |
| Vaccination - 1 dose | 38 | 207 | 0.3 | 0.1-0.8 | - | - | - | - |
|  |  |  |  |  |  |  |  |  |
| DOUBLE EXPOSURE |  |  |  |  |  |  |  |  |
| Vaccination - 2 doses | 17 | 1458 | 2.1 | 0.6-7.5 | 1.0 | N/A | - | - |
| Infection + Vaccination - 1 dose | 57 | 6542 | 9.6 | 4.4-20.8 | 4.5 | 1.9-10.4 | 1.0 | N/A |
|  |  |  |  |  |  |  |  |  |
| TRIPLE EXPOSURE |  |  |  |  |  |  |  |  |
| Infection + Vaccination - 2 doses | 18 | 11957 | 17.6 | 5.5-55.6 | 8.2 | 3.9-17.1 | 1.8 | 0.9-4.2 |

Table S3: Overall Antibody Levels (S1-RBD) by Sex and Study Group:

| Study Group | Males | | | | Females | | | |  |
| --- | --- | --- | --- | --- | --- | --- | --- | --- | --- |
|  | N | GMT | GMT Relative to NI | 95% CI | N | GMT | GMT Relative to NI | 95% CI | p-value (GMTs) |
| SINGLE EXPOSURE |  |  |  |  |  |  |  |  |  |
| Infection | 54 | 944 | 1.0 | N/A | 55 | 732 | 1.0 | N/A | 0.59 |
| Vaccination - 1 dose | 52 | 386 | 0.4 | 0.2-0.8 | 76 | 544 | 0.7 | 0.4-1.3 | 0.96 |
|  |  |  |  |  |  |  |  |  |  |
| DOUBLE EXPOSURE |  |  |  |  |  |  |  |  |  |
| Vaccination - 2 doses | 37 | 2562 | 2.7 | 1.4-5.3 | 46 | 2194 | 3.0 | 1.7-5.3 | 0.49 |
| Infection + Vaccination - 1 dose | 78 | 5111 | 5.4 | 3.4-8.6 | 124 | 4861 | 6.6 | 4.6-9.5 | 0.01 |
|  |  |  |  |  |  |  |  |  |  |
| TRIPLE EXPOSURE |  |  |  |  |  |  |  |  |  |
| Infection + Vaccination - 2 doses | 81 | 5336 | 5.6 | 3.4-9.3 | 81 | 5906 | 8.1 | 5.4-12.2 | 0.34 |

| Study Group | Males | | | | Females | | | |  |
| --- | --- | --- | --- | --- | --- | --- | --- | --- | --- |
|  | N | GMT | GMT Relative to NI | 95% CI | N | GMT | GMT Relative to NI | 95% CI | p-value (GMTs) |
| SINGLE EXPOSURE |  |  |  |  |  |  |  |  |  |
| Infection | 24 | 933 | 1.0 | N/A | 24 | 497 | 1.0 | N/A | 0.37 |
| Vaccination - 1 dose | 13 | 196 | 0.2 | 0.1-0.9 | 25 | 213 | 0.4 | 0.1-1.6 | 0.89 |
|  |  |  |  |  |  |  |  |  |  |
| DOUBLE EXPOSURE |  |  |  |  |  |  |  |  |  |
| Vaccination - 2 doses | 10 | 1244 | 1.3 | 0.3-5.6 | 7 | 1829 | 3.7 | 0.4-35.1 | 0.59 |
| Infection + Vaccination - 1 dose | 28 | 5775 | 6.2 | 2.1-18.7 | 29 | 7378 | 14.8 | 4.8-45.6 | 0.56 |
|  |  |  |  |  |  |  |  |  |  |
| TRIPLE EXPOSURE |  |  |  |  |  |  |  |  |  |
| Infection + Vaccination - 2 doses | 13 | 11854 | 12.7 | 4.1-39.3 | 5 | 12229 | 24.6 | 1.8-339.0 | 0.91 |

Table S4: Overall Neutralization Potential by Sex and Study Group:

| Study Group |  |  |  |  |  |  |  |  |
| --- | --- | --- | --- | --- | --- | --- | --- | --- |
|  | N | GMT | GMT Relative to VAX-2 | 95% CI | GMT Relative to HI-1 | 95% CI | p-value vs HI-1 | p-value vs VAX-2 |
| SINGLE EXPOSURE |  |  |  |  |  |  |  |  |
| Infection | 109 | 830 | - | - | - | - | - | - |
| Vaccination - 1 dose | 128 | 473 | - | - | - | - | - | - |
|  |  |  |  |  |  |  |  |  |
| DOUBLE EXPOSURE |  |  |  |  |  |  |  |  |
| Vaccination - 2 doses | 83 | 2351 | 1.0 | N/A | - | - | - | - |
| Infection + Vaccination - 1 dose | 202 | 4956 | 2.1 | 1.8-2.5 | 1.0 | N/A | - | <0.001 |
|  |  |  |  |  |  |  |  |  |
| TRIPLE EXPOSURE |  |  |  |  |  |  |  |  |
| Infection + Vaccination - 2 doses | 162 | 5614 | 2.4 | 1.9-3.0 | 1.1 | 0.9-1.3 | 0.10 | <0.001 |

Table S5: Comparing Overall S1-RBD Results to VAX-2 and HI-1:

| Study Group |  |  |  |  |  |  |  |  |
| --- | --- | --- | --- | --- | --- | --- | --- | --- |
|  | N | GMT | GMT Relative to VAX-2 | 95% CI | GMT Relative to HI-1 | 95% CI | p-value vs HI-1 | p-value vs VAX-2 |
| SINGLE EXPOSURE |  |  |  |  |  |  |  |  |
| Infection | 48 | 681 | - | - | - | - | - | - |
| Vaccination - 1 dose | 38 | 207 | - | - | - | - | - | - |
|  |  |  |  |  |  |  |  |  |
| DOUBLE EXPOSURE |  |  |  |  |  |  |  |  |
| Vaccination - 2 doses | 17 | 1458 | 1.0 | N/A | - | - | - | - |
| Infection + Vaccination - 1 dose | 57 | 6542 | 4.5 | 1.9-10.4 | 1.0 | N/A | - | 0.001 |
|  |  |  |  |  |  |  |  |  |
| TRIPLE EXPOSURE |  |  |  |  |  |  |  |  |
| Infection + Vaccination - 2 doses | 18 | 11957 | 8.2 | 3.9-17.1 | 1.8 | 0.9-3.8 | 0.11 | <0.001 |

Table S6: Comparing Overall Neutralization Results to VAX-2 and HI-1:

| Study Group (Ages 20-59 Only) |  | | | |
| --- | --- | --- | --- | --- |
|  | N | GMT | GMT Relative to NI | 95% CI |
| SINGLE EXPOSURE |  |  |  |  |
| Infection | 63 | 1000 | 1.0 | - |
| Vaccination - 1 dose | 102 | 406 | 0.4 | 0.3-0.6 |
|  |  |  |  |  |
| DOUBLE EXPOSURE |  |  |  |  |
| Vaccination - 2 doses | 26 | 3050 | 3.0 | 1.9-5.0 |
| Infection + Vaccination - 1 dose | 149 | 5177 | 5.2 | 4.1-6.5 |
|  |  |  |  |  |
| TRIPLE EXPOSURE |  |  |  |  |
| Infection + Vaccination - 2 doses | 75 | 5447 | 5.4 | 4.2-7.1 |

Table S7: S1-RBD Antibody Results in Individuals Between 20 and 59 Years Old:

| Study Group (Ages 20-59 Only) |  | | | |
| --- | --- | --- | --- | --- |
|  | N | GMT | GMT Relative to NI | 95% CI |
| SINGLE EXPOSURE |  |  |  |  |
| Infection | 29 | 799 | 1.0 | N/A |
| Vaccination - 1 dose | 23 | 108 | 0.1 | 0.1-0.4 |
|  |  |  |  |  |
| DOUBLE EXPOSURE |  |  |  |  |
| Vaccination - 2 doses | 3 | 3865 | 4.9 | 0.6-41.5 |
| Infection + Vaccination - 1 dose | 31 | 5942 | 7.4 | 3.0-18.2 |
|  |  |  |  |  |
| TRIPLE EXPOSURE |  |  |  |  |
| Infection + Vaccination - 2 doses | 3 | 7136 | 8.9 | 1.1-75.1 |

Table S8: Neutralization Results in Individuals Between 20 and 59 Years Old:

| Study Group (mRNA Vaccines Only) |  | | | |
| --- | --- | --- | --- | --- |
|  | N | GMT | GMT Relative to NI | 95% CI |
| SINGLE EXPOSURE |  |  |  |  |
| Infection | 109 | 830 | 1.0 | N/A |
| Vaccination - 1 dose | 100 | 542 | 0.7 | 0.4-1.0 |
|  |  |  |  |  |
| DOUBLE EXPOSURE |  |  |  |  |
| Vaccination - 2 doses | 71 | 2365 | 2.8 | 1.8-4.5 |
| Infection + Vaccination - 1 dose | 156 | 4918 | 5.9 | 4.3-8.1 |
|  |  |  |  |  |
| TRIPLE EXPOSURE |  |  |  |  |
| Infection + Vaccination - 2 doses | 129 | 5885 | 7.1 | 5.0-10.1 |

Table S9: S1-RBD Results in Individuals with mRNA Vaccines Only:

| Study Group (mRNA Vaccines Only) |  | | | |
| --- | --- | --- | --- | --- |
|  | N | GMT | GMT Relative to NI | 95% CI |
| SINGLE EXPOSURE |  |  |  |  |
| Infection | 48 | 681 | 1.0 | N/A |
| Vaccination - 1 dose | 31 | 218 | 0.3 | 0.1-0.8 |
|  |  |  |  |  |
| DOUBLE EXPOSURE |  |  |  |  |
| Vaccination - 2 doses | 17 | 1458 | 2.1 | 0.6-7.5 |
| Infection + Vaccination - 1 dose | 47 | 6397 | 9.4 | 4.0-21.9 |
|  |  |  |  |  |
| TRIPLE EXPOSURE |  |  |  |  |
| Infection + Vaccination - 2 doses | 18 | 11957 | 17.6 | 5.5-55.6 |

Table S10: Neutralization Results in Individuals with mRNA Vaccines Only:

Table S11: S1-RBD Results in Individuals Infected During Wave 1 (Ancestral):

| Study Group (Those Infected in Wave 1) |  | | | |
| --- | --- | --- | --- | --- |
|  | N | GMT | GMT Relative to NI | 95% CI |
| SINGLE EXPOSURE |  |  |  |  |
| Infection | 70 | 1236 | 1.0 | N/A |
| Vaccination - 1 dose | 128 | 473 | 0.4 | 0.3-0.6 |
|  |  |  |  |  |
| DOUBLE EXPOSURE |  |  |  |  |
| Vaccination - 2 doses | 83 | 2351 | 1.9 | 1.4-2.6 |
| Infection + Vaccination - 1 dose | 133 | 5325 | 4.3 | 3.4-5.5 |
|  |  |  |  |  |
| TRIPLE EXPOSURE |  |  |  |  |
| Infection + Vaccination - 2 doses | 125 | 5696 | 4.6 | 3.4-6.2 |

Table S12: Neutralization Results in Individuals Infected During Wave 1 (Ancestral):

| Study Group (Those Infected in Wave 1) |  | | | |
| --- | --- | --- | --- | --- |
|  | N | GMT | GMT Relative to NI | 95% CI |
| SINGLE EXPOSURE |  |  |  |  |
| Infection | 26 | 1122 | 1.0 | N/A |
| Vaccination - 1 dose | 38 | 207 | 0.2 | 0.1-0.5 |
|  |  |  |  |  |
| DOUBLE EXPOSURE |  |  |  |  |
| Vaccination - 2 doses | 17 | 1458 | 1.3 | 0.4-4.0 |
| Infection + Vaccination - 1 dose | 41 | 5614 | 5.0 | 2.0-12.6 |
|  |  |  |  |  |
| TRIPLE EXPOSURE |  |  |  |  |
| Infection + Vaccination - 2 doses | 15 | 13591 | 12.1 | 4.3-33.8 |

Table S13: Neutralization Results in Individuals Infected in Wave 2 (Alpha/Gamma):

| Study Group (Those Infected in Wave 2) |  | | | |
| --- | --- | --- | --- | --- |
|  | N | GMT | GMT Relative to NI | 95% CI |
| SINGLE EXPOSURE |  |  |  |  |
| Infection | 39 | 407 | 1.0 | N/A |
| Vaccination - 1 dose | 128 | 473 | 1.2 | 0.6-2.1 |
|  |  |  |  |  |
| DOUBLE EXPOSURE |  |  |  |  |
| Vaccination - 2 doses | 83 | 2351 | 5.8 | 3.1-10.6 |
| Infection + Vaccination - 1 dose | 62 | 4173 | 10.3 | 5.2-20.1 |
|  |  |  |  |  |
| TRIPLE EXPOSURE |  |  |  |  |
| Infection + Vaccination - 2 doses | 32 | 5264 | 12.9 | 5.3-31.7 |

Table S14: Neutralization Results in Individuals Infected in Wave 2 (Alpha/Gamma):

| Study Group (Those Infected in Wave 2) |  | | | |
| --- | --- | --- | --- | --- |
|  | N | GMT | GMT Relative to NI | 95% CI |
| SINGLE EXPOSURE |  |  |  |  |
| Infection | 22 | 378 | 1.0 | N/A |
| Vaccination - 1 dose | 38 | 207 | 0.5 | 0.2-1.9 |
|  |  |  |  |  |
| DOUBLE EXPOSURE |  |  |  |  |
| Vaccination - 2 doses | 17 | 1458 | 3.9 | 0.8-17.7 |
| Infection + Vaccination - 1 dose | 14 | 9963 | 26.4 | 5.6-124.8 |
|  |  |  |  |  |
| TRIPLE EXPOSURE |  |  |  |  |
| Infection + Vaccination - 2 doses | 2 | 4036 | 10.7 | 0.2-725.7 |

Table S15: S1-RBD Results in Nonhospitalized Individuals:

| Study Group (Nonhospitalized Individuals) |  | | | |
| --- | --- | --- | --- | --- |
|  | N | GMT | GMT Relative to NI | 95% CI |
| SINGLE EXPOSURE |  |  |  |  |
| Infection | 102 | 3495 | N/A | N/A |
| Vaccination - 1 dose | 128 | 3938 | 1.1 | 0.8-1.6 |
|  |  |  |  |  |
| DOUBLE EXPOSURE |  |  |  |  |
| Vaccination - 2 doses | 83 | 7637 | 2.2 | 1.5-3.2 |
| Infection + Vaccination - 1 dose | 197 | 8040 | 2.3 | 1.8-2.9 |
|  |  |  |  |  |
| TRIPLE EXPOSURE |  |  |  |  |
| Infection + Vaccination - 2 doses | 156 | 8634 | 2.5 | 1.9-3.2 |

Table S16: Neutralization Results in Nonhospitalized Individuals:

| Study Group (Nonhospitalized Individuals) |  | | | |
| --- | --- | --- | --- | --- |
|  | N | GMT | GMT Relative to NI | 95% CI |
| SINGLE EXPOSURE |  |  |  |  |
| Infection | 45 | 557 | N/A | N/A |
| Vaccination - 1 dose | 38 | 207 | 0.4 | 0.1-0.9 |
|  |  |  |  |  |
| DOUBLE EXPOSURE |  |  |  |  |
| Vaccination - 2 doses | 17 | 1458 | 2.6 | 0.8-9.0 |
| Infection + Vaccination - 1 dose | 54 | 6404 | 11.5 | 5.2-25.4 |
|  |  |  |  |  |
| TRIPLE EXPOSURE |  |  |  |  |
| Infection + Vaccination - 2 doses | 18 | 11957 | 21.5 | 6.9-66.3 |

Table S17: S1-RBD Results for Only Those Individuals with Neutralization Results:

| Study Group (Those with Neutralization Results) |  | | | |
| --- | --- | --- | --- | --- |
|  | N | GMT | GMT Relative to NI | 95% CI |
| SINGLE EXPOSURE |  |  |  |  |
| Infection | 48 | 1111 | N/A | N/A |
| Vaccination - 1 dose | 38 | 420 | 0.4 | 0.1-1.2 |
|  |  |  |  |  |
| DOUBLE EXPOSURE |  |  |  |  |
| Vaccination - 2 doses | 17 | 3528 | 3.2 | 0.9-11.5 |
| Infection + Vaccination - 1 dose | 57 | 5704 | 5.1 | 2.4-10.9 |
|  |  |  |  |  |
| TRIPLE EXPOSURE |  |  |  |  |
| Infection + Vaccination - 2 doses | 18 | 7405 | 6.7 | 2.1-21.1 |
